# Protocol for a cluster randomized control trial of the impact of the *Breaking the Man Code* workshops on adolescent boys’ intentions to seek help

**DOI:** 10.1186/s13063-022-06034-0

**Published:** 2022-02-03

**Authors:** Kylie King, Marisa Schlichthorst, Patty Chondros, Simon Rice, Anna Clark, Long Khanh-Dao Le, Cathrine Mihalopoulos, Jane Pirkis

**Affiliations:** 1 Turner Institute for Brain and Mental Health, Monash University, Melbourne, Australia; 2 Centre for Mental Health, Melbourne School of Population and Global Health, The University of Melbourne, Melbourne, Australia; 3 Department of General Practice, Melbourne Medical School, The University of Melbourne, Melbourne, Australia; 4 Orygen, Parkville, Melbourne, Australia; 5 Centre for Youth Mental Health, The University of Melbourne, Melbourne, Australia; 6 Deakin Health Economics, Institute for Health Transformation, School of Health and Social Development, Deakin University, Melbourne, Australia

**Keywords:** Help-seeking, Masculinity, Suicide, Youth, Mental health, Trial, Cluster

## Abstract

**Background:**

Males in Australia and many other countries account for three-quarters of all deaths by suicide. School-based programs to support young men’s wellbeing have become increasingly common in recent years and show much promise to tackle the issue of male suicide by fostering protective factors and mitigating harmful factors. However, only a few of these programs have been evaluated. This trial seeks to address the lack of knowledge about the potential for school-based gender-transformative programs to have a positive impact on boys. *Breaking the Man Code* workshops, delivered by *Tomorrow Man* in Australia, challenge and transform harmful masculinities with young men with a view to ultimately reducing their suicide risk. The trial aims to examine whether adolescent boys who participate in the *Breaking the Man Code* workshop demonstrate an increase in their likelihood of seeking help for personal or emotional problems compared to boys waiting to take part in the workshop.

**Methods:**

A stratified cluster randomized controlled superiority trial with two parallel groups will be conducted. Schools will be randomly allocated 1:1, stratified by location of the schools (rural or urban), state (Victoria, NSW, or WA), and mode of workshop delivery (face-to-face or online), to the intervention group and waitlist control group.

**Discussion:**

The *Breaking the Man Code* workshops show great promise as a school-based prevention intervention. The trial will fill a gap in knowledge that is critically needed to inform future interventions with boys and men. Some methodological challenges have been identified related to the COVID-19 pandemic in Australia, such as delays in ethics approvals and the need for *Tomorrow Man* to introduce an online delivery option for the workshop. The trial protocol has been designed to mitigate these challenges. The findings from the trial will be used to improve the workshops and will assist others who are designing and implementing suicide prevention interventions for boys and men.

**Trial registration:**

Australian New Zealand Clinical Trials Registry (ACTRN12620001134910). Registered on 30 October 2020

## Administrative information

Note: the numbers in curly brackets in this protocol refer to SPIRIT checklist item numbers. The order of the items has been modified to group similar items (see http://www.equator-network.org/reporting-guidelines/spirit-2013-statement-defining-standard-protocol-items-for-clinical-trials/).

**Table:** 

• Title {1}	A cluster randomized controlled trial of the impact of the '*Breaking the Man Code*' workshops on adolescent boys' intentions to seek help
Trial registration {2a and 2b}.	ANZCTRN 12620001134910 U1111-1253-6472
Protocol version {3}	01/April/2021 Version 1
Funding {4}	Australian Rotary Health Australian Government Medical Research Fund
Author details {5a}	Kylie King, PhD (Turner Institute for Brain and Mental Health, Monash University) Marisa Schlichthorst, PhD (Centre for Mental Health, Melbourne School of Population and Global Health, The University of Melbourne) Patty Chondros, PhD (Department of General Practice, The University of Melbourne) Simon Rice, PhD (Orygen, Parkville, Australia; Centre for Youth Mental Health, The University of Melbourne) Anna Clark, M.Psych (Turner Institute for Brain and Mental Health, Monash University) Long Khanh-Dao Le, PhD (Deakin Health Economics, Institute for Health Transformation, School of Health and Social Development, Deakin University) Cathrine Mihalopoulos, PhD (Deakin Health Economics, Institute for Health Transformation, School of Health and Social Development, Deakin University) Jane Pirkis, PhD (Centre for Mental Health, Melbourne School of Population and Global Health, The University of Melbourne
Name and contact information for the trial sponsor {5b}	Turner Institute for Brain and Mental Health Monash University 18 Innovation Walk, Clayton, VIC, Australia, 3800 + 61 3 99052389 kylie.king@monash.edu
Role of sponsor {5c}	The Turner Institute of Brain and Mental Health at Monash University, will host the trial and the Principal Investigator Dr King who will oversee the study design, data collection, management, analysis, and interpretation of data, writing of the report, and the decision to submit the report for publication and will have ultimate authority over these activities. The funders will have no role or authority in these processes.

## Introduction

### Background and rationale {6a}

Males in Australia and many other countries account for three-quarters of all deaths by suicide [1, 2]. In Australia, the rates of suicide among males are higher than those of females across all age groups [1]. Among young Australians, rates of suicide and suicide-related behavior remain high despite a national priority for the prevention of youth suicide. The suicide of a young person has a devastating ripple effect on friends, family, and the community [3].

The higher rate of suicide among males has been attributed to several factors including lethality of means, substance use and associated impulsivity, reduced social connectedness, reduced help-seeking, and—perhaps underpinning these factors—conformity to some masculine norms [4–7].

While some masculine norms have positive impacts on wellbeing [8], conformity to masculine norms such as self-reliance has been linked to increased suicidal ideation and behavior [9, 10]. This may be because some masculine norms act to discourage seeking help for emotional problems, which is perceived as weakness or failure [11–13]. These masculine norms are laid down early in life with men reporting conformity to these norms from adolescence [14, 15]. Given the gendered nature of suicide and the role of masculine norms, a gendered focus that attends to the social context of suicide experienced by men has long been recommended for suicide prevention interventions [16, 17]. The fact that gender role socialization starts early in life suggests the need for early interventions with adolescent males to ameliorate negative impacts of rigid adherence to masculine norms. Reduction in self-reliance in particular, and increases in help-seeking, may ultimately reduce the rate of suicide among men.

Despite the strong case for gendered programs and the importance of the social context for male suicide risk, there is a limited knowledge base regarding the use of such programs [18]. Few suicide prevention interventions have specifically targeted males, and fewer still have focused on the role of masculine norms as a determinant of suicide. A scoping review of men’s suicide prevention published in 2017 identified only six interventions targeting men [19].

School-based programs designed to specifically support young men’s wellbeing have become increasingly common in recent years. In Australia, *Tomorrow Man*’s *Breaking the Man Code* workshops (tomorrowman.com.au), the *Man Cave* program (themancave.life), and Menslink’s *Silence is Deadly* program (menslink.org.au/silence-is-deadly) are three examples. They each adopt an upstream approach to male suicide prevention by focusing on reducing conformity to harmful masculine norms and increasing help-seeking. However, the evidence base for the effectiveness of gendered school-based programs to bring about these and other positive impacts for young men is lacking. A systematic review of young men’s wellbeing programs by Gwyther and colleagues, published in 2019, found 40 studies [20]. Of which, only four of these addressed harmful aspects of masculinity to bring about positive changes for young men (labeled “gender transformative”), and a further 10 tailored content to young men, but did not explicitly address conformity to harmful masculine norms (“gender sensitive”). Each of these interventions reported some positive impacts but no changes in any variables related to masculinity. The review concluded with overall support for the effectiveness of programs for boys and young men, particularly via gendered interventions and school-based environments. However, it cautioned that many programs remain unevaluated and that there is a need to determine which approaches work best. Another recent review by Calear and colleagues explored psychosocial interventions for youth suicide [21]. This review identified 28 trials and found that just over half of the programs had an impact on reducing suicidal ideation, suicide attempts, or self-harm. However, none of the trials focused exclusively on young males or attended to masculine norms. The authors concluded that further research is needed to strengthen the evidence base for youth suicide prevention interventions. Like Gwyther and colleagues, Calear and colleagues highlighted the potential for school-based programs. Recently, Calear and colleagues have published the findings of their trial of the *Silence is Deadly* program, an upstream public health intervention for increasing help-seeking intentions for mental disorders and suicide for adolescent males. This gender transformative program was found to significantly increase help-seeking intentions from friends [22].

In summary, school-based gender-transformative programs are showing some promise as an upstream approach to male suicide prevention; however, more research is needed to confirm impacts for participants and understand more about the process by which impacts are achieved.

This trial seeks to address current knowledge gaps in the potential for school-based gender-transformative programs to have a positive impact on the help-seeking intentions of adolescent boys. Between 2017 and 2021, *Tomorrow Man* has delivered over 750 *Breaking the Man Code* workshops that challenge and transform harmful masculinities with Australian adolescent males with a view to ultimately reducing their suicide risk around Australia. However, these workshops have not been formally evaluated in terms of their impact on participants. Findings about the impact of the workshops will provide lessons for suicide prevention programs that focus on transforming potentially harmful masculine norms to bring about positive mental health outcomes for young males.

This trial aims to determine the impact of *Breaking the Man Code* workshops on adolescent boys.

### Objectives {7}

The objective of the trial is to determine the impact of the *Breaking the Man Code* workshops on adolescent boys’ (in year 10, 11, or 12) intentions to seek help for a personal emotional problem, conformity to masculine norms, depression risk, perceived social support, and quality of life.

### Trial design {8}

This study is a stratified cluster randomized controlled trial with two parallel groups. Schools will be randomized to the intervention group and waitlist control group with a 1:1 allocation stratified by location of the schools (rural or urban), state (Victoria, NSW, or WA), and mode of workshop delivery (face-to-face or online),

## Methods: participants, interventions, and outcomes

The trial methods are reported using the SPIRIT reporting guidelines as shown in the checklist [23].

### Study setting {9}

The trial will be conducted within secondary schools in Australia in New South Wales, Victoria, and Western Australia. Recruitment of schools began in late 2020. These three states were chosen as they are the states of Australia where Tomorrow Man undertakes most of their work. There will be a mix of rural, urban, private, and public schools. All schools in these three states who receive a *Breaking the Man Code* workshop within the trial period are eligible to take part (subject to relevant ethics approvals) and will be invited to participate. Recruitment of students into the study began in the second school term of 2021 (April). Data collection will occur in the 2021 and 2022 school years.

### Eligibility criteria {10}

Eligible participants will be male senior secondary school students in year 10, 11, or 12 who are enrolled to take part in a *Breaking the Man Code* workshop in 2021 or 2022 within their school and have parental/guardian informed consent.

School inclusion:

1. Request a *Breaking the Man Code* workshop for their year 10, 11, or 12 males in 2021 or 2022.
2. Agree to schedule the workshop within either the intervention period or the waitlist period (after the trial is complete) as instructed by the researchers.
3. Agree to distribute study information to parents of boys enrolled in the workshop and to allocate two class times, 6–8 weeks apart, for students to complete the baseline and follow-up questionnaire.

Student inclusion:

1. Student within a participating school in years 10, 11, or 12
2. Self-identified male
3. Enrolled to take part in a *Breaking the Man Code* workshop
4. Parent has provided consent for them to take part in the trial

There are no exclusion criteria.

### Who will take informed consent? {26a}

The principals of participating schools will provide consent for school participation. Parental (or guardian) consent will be sought in all schools. The method of parental consent will be determined in collaboration with each school. To maximize response, the method will ideally be the same method of consent that each school uses for their other student activities (such as excursions and incursions). This will likely involve using school digital platforms (such as Daymap.net) or sending emails to parents with links to online consent procedures. Hard copies of study information and consent forms can be sent home with students, for return to the researchers in a provided prepaid envelope, for those without online access. Parents or guardians will be asked to provide their son’s school name and school email address which will be used by an independent data management company (Logicly) to provide students with the Plain Language Statement, consent form, and a link to the online survey once parental consent is received. Students whose parents have consented to their participation will be asked for their informed assent online within the allocated class time at school after reading the Plain Language Statement.

### Additional consent provisions for collection and use of participant data and biological specimens {26b}

Not applicable, no biological specimens collected.

## Interventions

### Explanation for the choice of comparators {6b}

The trial will compare participants who receive the *Breaking the Man Code* workshop within their school with those who are waitlisted to receive the workshop and receive only their usual school curriculum. These conditions were chosen so that no school would be denied the opportunity to receive the intervention, as both groups will have shown an interest in receiving the workshops. It is expected that the two groups of schools (intervention and waitlist) will be similar given that they are taken from the same population (schools requesting a workshop).

### Intervention description {11a}

The intervention group will receive the *Breaking the Man Code* workshop. This workshop will be delivered in class time at school by a *Tomorrow Man* trained facilitator. The workshops are delivered over 2 h in a face-to-face or an online format (i.e., via a video meeting platform) to groups of 30–35 male students. Prior to the COVID-19 pandemic, workshops were delivered face-to-face only. In response to the pandemic, and associated lockdowns and travel restrictions, the online mode was developed in 2020 during the design phase of the trial. The workshop content is similar in both delivery modes, with small adjustments. The mode of delivering is now generally chosen based on school preference, facilitator availability (particularly when the facilitator is required to travel to rural areas), and, where relevant, COVID restrictions. One facilitator usually provides all the workshops within one school, with up to six facilitators delivering the workshops across schools nationally. The average number of workshops per school is three, with an average of 33 students per workshop.

The interactive *Breaking the Man Code* workshops facilitate honest and authentic conversations with year 10, 11, and 12 male students, in order to define a masculinity that “generates purpose, pride, and health for the men of today and tomorrow.” The workshops aim to develop protective factors such as positive attitudes towards help-seeking, emotional expressiveness, and social support. The workshop involves discussion about the stereotypes of what it means to be a man in Australia today, and the impacts that these can have on men’s mental health. It explores the (unwritten) rules that exist for men on how to behave (the “man code”) and investigates why men and boys feel obliged to live up to these expectations and stereotypes. The workshop then unpacks the impact of the “man code” on boys and men and allows boys to share their experience living with the stereotypes while encouraging them to question norms and redefine their own rules.

### Criteria for discontinuing or modifying allocated interventions {11b}

There will be no modification to the interventions. Schools and participating students will be free to withdraw from the trial at any point. This will not impact on their involvement in the workshop. Students who choose not to take part in, or complete, the trial will still be able to take part in the workshop. Facilitators will monitor students for signs of distress during the workshop and the decision to withdraw students from the workshop will be made collaboratively between the facilitators and the schools. The researchers will not be involved in this process.

### Strategies to improve adherence to interventions {11c}

All students who complete the baseline questionnaire will be invited to complete the follow-up questionnaire, regardless of whether or not they attend the workshop (e.g., due to illness). Logicly (the independent data manager sub-contractor) will monitor parental consent, student assent, and student survey completion rates per school while maintaining allocation blinding and will report this to the researchers weekly. An Advisory Group, comprising experts in men’s and youth mental health, and a Consumer Reference Panel, comprising young males who have previously received the workshop, are supporting the trial. These groups will provide advice regarding strategies to improve participant adherence.

### Relevant concomitant care permitted or prohibited during the trial {11d}

There are no restrictions on concomitant care.

### Provisions for post-trial care {30}

Any participants who suffer harm, such as psychological distress, from the trial will be supported by their school’s welfare processes.

### Outcomes {12}

#### Primary outcome

Difference between the intervention and waitlist control groups at follow-up in the mean change in intentions to seek help as measured by an adapted version of the General Help Seeking Questionnaire (GHSQ) [24] at 8 weeks post-baseline.

#### Secondary outcomes

Difference between the two study groups at 8 weeks post-baseline in:

1. Mean change in conformity to masculine norms as measured by the Conformity to Masculine Norms Inventory (CMNI-22) [25].
2. Mean change in depression risk as measured by the Male Depression Risk Scale Short Form [26].
3. Mean change in perceived social support as measured by the Modified Medical Outcomes Study Social Support Survey (MOS-SS) Emotional/Informational support subscale [27]
4. Mean change in quality of life as measured by the Child Health Utility Instrument (CHU-9D) [28]

#### Economic outcomes

The economic outcome of the intervention will be determined in comparison to the waitlist control group from a health care perspective and a partial societal perspective. The CHU-9D data will also enable the calculation of quality-adjusted life years (QALYs) that will be used in a cost-utility analysis. Key costs and their measure(s) include (i) intervention costs, (ii) parents’ productivity losses (based on the number of days their son is absent from school), and (iii) health care service costs during the trial follow-up period (collected using a modified Resource Utilization Questionnaire (RUQ) [29]).

### Participant timeline {13}

**Fig. 1:**
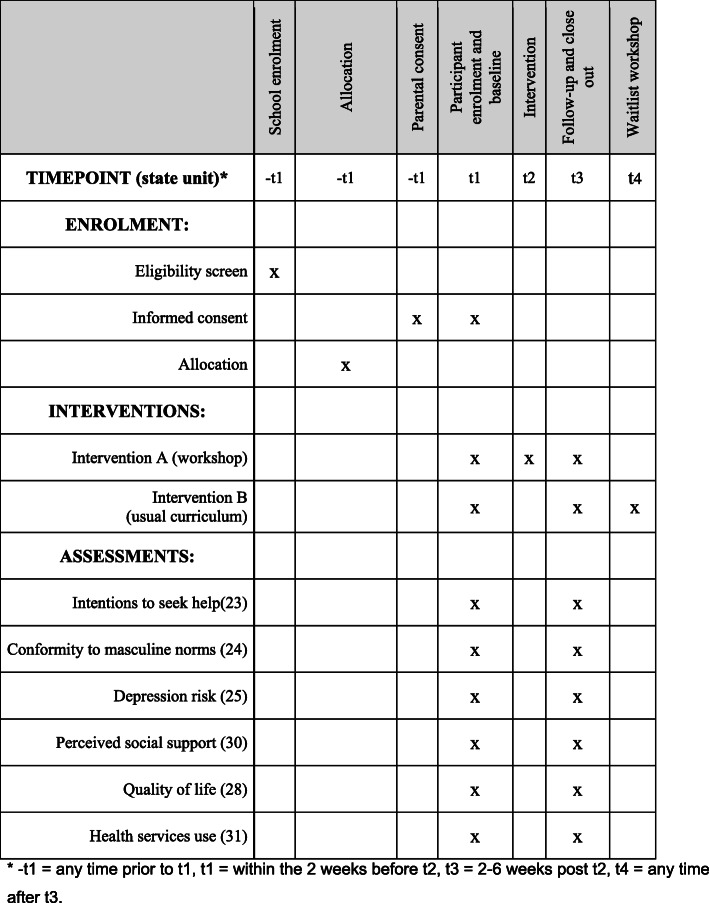
Schedule of enrolment, interventions, and assessments

The participant timeline is shown in Fig. 1.

From the end of 2020 and throughout 2021 and 2022, as schools contact *Tomorrow Man* to book in workshops for the following year, *Tomorrow Man* will undertake a school eligibility check with interested schools (i.e., to ensure it meets the trial inclusion criteria). Contact details of eligible schools will be forwarded to the researchers who will provide schools with information about the trial and ask school principals to give their consent for the schools to take part. The researchers will randomize the school to the intervention or waitlist group.

All schools will then be sent a Plain Language Statement and parental consent form for all parents of eligible students. Schools will distribute this information to parents, who will be asked to provide consent for their son’s participation. Students whose parents have consented will receive an email informing them that their parents have consented to their participation in the trial and that shortly they too will receive further information. This email will allow checks for “bounce-backs” and confirm any email errors with parents prior to student participation.

For schools in the intervention group, soon after randomization, schools will schedule a class time for all the male students who are enrolled to take part in the workshop. This class time will be within the 2 weeks prior to the workshop. The school will advise the researchers of this class time and the researchers will direct Logicly to send a second email to students with parental consent. The email will contain a link to the Plain Language Statement, assent form, and baseline questionnaire. The school staff member will activate the baseline questionnaire within the class time using a link provided to them by Logicly. After reading the Plain Language Statement and providing their assent, the students will have access to complete the online survey which will take about 20 min. Intervention group schools will then receive the workshop within the following 2 weeks. Schools will be asked to schedule a second-class time with students 4 to 6 weeks after students received the workshop to administer the follow-up questionnaire. Prior to this class, students who completed the baseline questionnaire will receive an email containing a link to the deactivated online follow-up questionnaire. Within the class time, a staff member will direct students to the email, if they have received it. The staff member will activate the follow-up questionnaire and the students will complete it

For schools in the waitlist group, soon after randomization, the schools will schedule a class time for all the male students who are enrolled to take part in the workshop. The school will advise the researchers of this class time and the researchers will direct Logicly to send an email to students with parental consent containing a link to the Plain Language Statement, assent form, and baseline questionnaire. The school staff member will activate the baseline questionnaire within the class time using a link provided to them by Logicly. After reading the Plain Language Statement and providing their assent, the students will have access to complete the online survey which will take about 20 min. The schools will then provide school as usual for the students over the following weeks. Schools will be asked to schedule a second class time for students to complete the follow-up questionnaire 6 to 8 weeks after the baseline questionnaire. The staff member will activate the questionnaire and the students will complete it. They will receive the workshop after the second survey has been completed by the students.

In either group, if a student has completed the baseline questionnaire, but cannot locate the email for the follow-up questionnaire, then an automated process will enable the teacher to provide the student email to Logicly who will resend the email.

If schools or individual participants withdraw from the trial at any time (prior to baseline or follow-up questionnaire assessment), the data provided by participants up to that point will be included in the trial.

### Sample size {14}

For 90% power and a two-sided 5% significance level, a sample of 1000 participants across 40 schools (a minimum of 25 students on average per school) will be required to detect a standardized effect size of 0.3 in mean change in GHSQ score between the study groups. This is equivalent to a 3-point difference in mean GHSQ score between the study groups (standard deviation of 10), which was found in the recent Man Up trial with adult men [30]. Sample size accounts for the correlation of outcomes within schools of 0.03 [31] and assumes 20% attrition at follow-up of students and allows for the potential loss of two schools over the period of the study.

### Recruitment {15}

Five school terms (terms 2, 3, and 4 in 2021 and terms 2, 3, and 4 in 2022) have been provided for data collection from the students. The desired sample size of 1000 participants across 40 schools is feasible within *Tomorrow Man*’s forecast of approximately 5000 male student participants in 150 workshops across Australia in the study period.

## Assignment of interventions: allocation

### Sequence generation {16a}

Schools will be randomized in a 1:1 ratio to the intervention or control group using an allocation sequence stratified by location of the schools (rural or urban), state (Victoria, NSW, or WA), and mode of workshop delivery (face-to-face or online), generated using a biased-coin algorithm [32]. The imbalance tolerance for the biased-coin algorithm will not be disclosed until all the schools have been randomized to ensure concealment.

### Concealment mechanism {16b}

*Tomorrow Man* will invite schools to the trial and then will pass the details of interested schools onto one of the researchers. This researcher will obtain school consent and will collect details including school location, state, and mode of workshop delivery. The researcher will then pass this deidentified information to the trial statistician who will randomly allocate each school to either the intervention or the waitlist group. The statistician will advise the researcher of the allocation who will then re-identify the school. The rest of the research team, including the statistician, will remain blinded to the identity of schools until data analysis is complete. *Tomorrow Man* workshop facilitators will be delivering workshops throughout the year to schools that are participating in the trial, as well as to schools that are not participating. *Tomorrow Man* will not tell facilitators which schools are participating. Parents and students in schools in each group will not be aware of the presence of the alternative group and will not be informed that their participation is to evaluate the *Breaking the Man Code* workshops. They will be told that the study is about male student wellbeing.

### Implementation {16c}

Online questionnaire data collection will be undertaken by Logicly (the data management subcontractor) who will use a purpose-built online platform for the survey. They will generate unique URLs for each participant based on their email address, so that participant responses can be connected across the two questionnaires. At the end of the trial, Logicly will provide the questionnaire data to the researchers in a deidentified, blinded format. Unblinding of the groups will occur after data analysis.

## Assignment of interventions: blinding

### Who will be blinded {17a}

The students participating in the trial, the workshop facilitators, the statistician, and the researchers not involved in randomization will be blinded to the names and allocation of schools. Schools will know their allocation based on the timing of the workshop relative to the questionnaires but will be asked not to inform students or workshop facilitators. *Tomorrow Man* workshop facilitators will be blinded, as they will not know which schools are participating in the trial.

### Procedure for unblinding if needed {17b}

If a school or participant withdraws from the trial, unblinding will be required to ensure that participants are not contacted for the next stage of the trial (e.g., the baseline or follow-up questionnaire). Schools will advise the researchers who will then advise Logicly. Participants can notify the researchers via the trial email address (that is monitored by the researcher who was involved in randomization) provided on the Plain Language Statement. The researchers will seek to determine the reason for school or participant withdrawal.

## Data collection and management

### Plans for assessment and collection of outcomes {18a}

Outcome measures will be collected from students at baseline after randomization of schools. For the intervention group, data will be collected at baseline within 2 weeks of the intervention being delivered. The follow-up questionnaire will be administered 4–6 weeks after the workshop. For the waitlist control group, baseline and follow-up data collection will occur 6–8 weeks apart before the workshop is delivered.

For both groups, the baseline and follow-up questionnaires will be administered online within an allocated class time. The baseline questionnaire will include demographic questions and the measures related to the primary, secondary, and economic outcomes that are described below. The follow-up questionnaire will be a repeat of the baseline questionnaire, minus the demographic questions, with the addition of some open-ended questions for the intervention group.

#### Demographic information

Five demographic questions will be asked: age (in years); gender (male, transgender male, non-binary/gender diverse, don’t know, prefer not to say, or participant described); sexual orientation (gay or homosexual, straight or heterosexual, bisexual, something else, do not know, prefer not to say); language mainly spoken at home (English, Italian, Greek, Cantonese, Arabic, Mandarin, Vietnamese, other (specify)); and Aboriginal or Torres Strait Islander status (No, Aboriginal, Torres Strait Islander, both).

#### Primary outcome

Intentions to seek help for a personal or emotional problem will be measured by the General Help-seeking Questionnaire (GHSQ) [24]. The GHSQ has been found to be reliable and valid and to be a suitable measure of help-seeking intentions in a range of contexts, and the scale can be modified to add extra response items. The GHSQ asks participants: “If you were having a personal or emotional problem, how likely is it that you would seek help from the following people or services?” Response options include an intimate partner, friend, or doctor. Participants respond on a 7-point Likert scale (1 = extremely unlikely to 7 = extremely likely). The following additional response options will be included: online health chat rooms, online searches for health information, social media, and someone at school. The final score is the sum of responses to the 10 items (range 10–70), where higher scores indicate higher intentions to seek help.

#### Secondary outcomes

Conformity to norms of masculinity will be assessed via the Conformity to Masculine Norms Inventory (CMNI-22), and the 22-item scale will assess participants’ conformity to 11 potentially harmful masculine norms: emotional control, risk-taking, violence, dominance, playboy, self-reliance, primacy of work, power over women, heterosexual presentation, physical toughness, and pursuit of status [25]. Items are answered on a 4-point Likert scale (0 = strongly disagree to 3 = strongly agree), with the sum of the item scores providing a range between 0 and 66, where higher scores indicate higher conformity to masculine norms. The CMNI-22 has been found to have good internal consistency, criterion validity, and test-retest reliability [25, 33]. Recently, this scale has been revised and updated to a 30-item version with improved psychometrics [34]. However, the 22-item scale will be used in this trial in the interest of brevity for adolescent participants and to allow comparison with data from the Ten to Men study (The Australian Longitudinal Study of Male Health being undertaken by the Australian Institute of Family Studies https://tentomen.org.au).

Depression risk will be assessed using a modified 7-item form of the 22-item Male Depression Risk Scale (MDRS-Short Form) [26]. The paper reporting the properties of the modified short scale is under preparation (Hereen et al: Development and Validation of the MDRS Short Form for Clinical Use, in preparation). This 7-item scale has been chosen to capture the externalizing symptoms of depression common to males: emotional suppression, drug use, alcohol use, anger and aggression, somatic symptoms, and risk taking. Items are answered on a 5-point Likert scale (0 = none of the time to 4 = all of the time), and the total score for depression risk is the sum of the 7 item scores, ranging between 0 and 28. Higher scores indicate a higher depression risk. The 22-item scale reports satisfactory psychometric properties and the 7-item scale correlates strongly with the larger scale and has good internal consistency and test-re-test reliability and excellent CFA model fit [35, 36].

Perceived social support will be assessed using the emotional/informational subscale of the Medical Outcomes Study Social Support Survey (MOS-SS) [27]. This 8-item subscale asks participants about the kind of emotional and informational support available to them, such as someone: to turn to for suggestions about how to deal with a personal problem, who understands your problems, and to give you good advice about a crisis. The items are answered using a 5-point Likert scale (1 = none of the time to 5 = all of the time). The sum of the item scores provides a total score ranging between 8 and 40 where higher scores reflect higher levels of perceived support. The scale has been found to have good psychometric properties among young non-clinical populations [37].

Quality of life will be assessed using the Child Health Utility Instrument (CHU-9D) [38]. This 9-item scale asks participants about their functioning today across domains of worry, sadness, pain, tiredness, annoyance, school, sleep, daily routine, and activities. The items are answered using a 5-point Likert scale (1 = I do not feel X today to 5 = I feel very X today). The CHU9D has been recommended for measuring the quality of life in adolescent populations in Australia [39].

#### Economic outcomes

The CHU9D will also be used to derive health state utility values by using the previously published value set, reflecting Australian adolescents’ preferences, to derive quality-adjusted life years (QALYs) [40].

Health service use will be assessed using a modified Resource Utilization Questionnaire (RUQ) [29]. This 21-item questionnaire was developed to measure the health services and costs of health care. This questionnaire has been modified from the version used in the Young Minds Matter Survey to enable self-report by adolescents and will ask which health professionals participants have seen in the past 4 weeks for their mental health (number of the visits, location, and costs of visits), and medications taken for emotional or behavioral concerns in the past 4 weeks (type, dosage), and days of missed school [28]. Resource use and unit costs will be combined to derive a total cost for each participant.

#### Participant’s perception and impact of the intervention

In addition to these standardized measures, in the follow-up questionnaire, the intervention group will be asked a set of closed and open-ended questions regarding their perceptions of the workshop (What parts did you like the most/least? Did you change your attitudes or behaviors in any of the following ways?) and the impact of the workshop on their lives (Did the workshop change your ideas about what sort of man you’d like to be? Did the workshop change the way you talk with your mates/family about personal stuff?).

The questionnaires are available from the principal investigator.

### Plans to promote participant retention and complete follow-up {18b}

The study Advisory Group, Consumer Reference Panel, and other researchers who are working in school-based research will be consulted with regarding strategies to enhance retention of schools and students in the trial. Research that ensures the quality and impact of the *Breaking the Man Code* workshops will likely be appealing to schools who receive the workshops. Schools will also be provided with a one-off payment of A$800 after their involvement in the trial to compensate for the administrative burden. This payment will act to reduce any barriers to school participation. Students will not be incentivized or coerced to take part as it is the requirement of the ethics approvals. The Plain Language Statement will provide information to students about the benefits of participation.

### Data management {19}

All data will be self-report and entered by students into a secure online questionnaire that will be managed by Logicly. The questionnaires will be online and programmed with a logic that minimizes missing data by alerting participants to unanswered questions and maximizes data quality (e.g., range checks for data values). At the end of the data collection period, all data will be provided in the deidentified form to the researchers. Data will be stored in accordance with Monash University data storage policies [41].

### Confidentiality {27}

During the trial, the data will be stored by Logicly. Data will only be stored in electronic form and will be held securely on password-protected computers. Once data collection is finished, deidentified data will be provided to the researchers named on this protocol. Any hard copy printouts of data will be held in locked filing cabinets in locked offices. At the end of the project, deidentified coded quantitative data collected in the questionnaires will be uploaded to the Monash University Research Repository to be held for a maximum of 5 years after the last publication from the trial researchers. Individual questionnaire responses will be aggregated for analysis and reporting. No identifying information about schools or participants will be included when reporting findings from the trial.

### Plans for collection, laboratory evaluation, and storage of biological specimens for genetic or molecular analysis in this trial/future use {33}

Not applicable, no biological samples were collected.

## Statistical methods

### Statistical methods for primary and secondary outcomes {20a}

Descriptive statistics will be used to summarize school and participant characteristics between study groups. Analysis will use an intention to treat (ITT) approach, where participants will be analyzed in the study group to which they were randomized. Statistical analysis will be conducted using Stata Software 15 [42].

For the primary analysis, linear mixed effect models will be used to estimate the difference in mean change in GHSQ scores at follow-up between the study groups, with random effects for school and fixed effects for study group, baseline GHSQ scores, and stratification factors (location of the schools, state, and mode of workshop delivery). The estimated intervention effect will be reported as the difference in mean GHSQ scores between intervention and control groups, with 95% confidence interval and *p*-value. Similar analysis will be undertaken for secondary outcomes (conformity to masculine norms, depression risk, perceived social support, and quality of life).

#### Economic evaluation

Generalized linear models will be used to assess mean difference in costs and QALYs in the two study groups. For both groups, mean values of costs and QALYs will be reported, as well as mean differences between the groups. An incremental cost-effectiveness ratio (ICER) will be calculated as the difference in average cost between the groups, divided by the difference in average QALYs. Uncertainty in the data will be handled by using nonparametric bootstrapping from the distribution of the observed cost/QALY pairs (e.g., 1000 simulated replications) to determine confidence intervals (CIs) and presented in a cost-effectiveness plane along with a cost-effectiveness acceptability curve.

#### Participant’s perception and impact of the intervention

Descriptive statistics and inductive thematic analysis will be used to analyze responses to the closed and open-ended questions about participants’ perceptions and the impact of the workshop.

### Interim analyses {21b}

No interim analysis will be conducted.

### Methods for additional analyses (e.g., subgroup analyses) {20b}

Secondary analyses will adjust for pre-specified baseline variables in the regression models for primary and secondary outcomes. Exploratory analyses will include examining for effect modification on the outcomes between rural vs urban location of the schools, mode of workshop delivery, age, language spoken at home, gender, sexual orientation, and Aboriginal or Torres Strait Islander status.

To explore the robustness of the cost-effectiveness results, sensitivity analyses will be carried out. Both complete case analysis and intention to treat analysis will be conducted. The parameters including intervention costs or intervention pathway or proportion of adolescent males who receive the intervention will be varied in the sensitivity analysis.

### Methods in analysis to handle protocol non-adherence and any statistical methods to handle missing data {20c}

Non-compliance to the assigned intervention on the estimated intervention effect will be investigated using complier average causal effect (CACE) analysis for the primary and secondary outcomes [43].

Appropriate methods for handling the missing data will be informed by a blinded review of the data. Information on reasons for not completing the surveys and/or workshops (e.g., absent on the day, did not assent) will be collected for the students whose parents consent to the trial. Information will also be collected on the number of students who attended the workshops from Tomorrow Man. Sensitivity analyses using a pattern-mixture model will be used to assess the robustness of the missing data assumption for the primary outcome.

A statistical analysis plan detailing the additional analyses, including CACE analysis, approach for handling missing data, and sensitivity analyses, will be made available prior to the statistical analysis of the primary outcome.

### Plans to give access to the full protocol, participant-level data, and statistical code {31c}

At the end of the project, deidentified coded quantitative data collected in the questionnaires will be uploaded to the Monash University Research Repository to be held for a maximum of 5 years after the last publication from the trial researchers. Qualitative responses will not be uploaded to this repository due to the potential to identify participants. Access to the data will be determined by the researchers on a case-by-case basis and only if there is a detailed research plan and relevant ethics approvals. The study protocol is available at the Australian New Zealand Clinical Trials Registry. The full protocol and statistical code will be available by contacting the principal investigator.

## Oversight and monitoring

### Composition of the coordinating center and trial steering committee {5d}

There is no formal committee for the coordination and oversight of this trial. The principal investigator will oversee and coordinate all aspects of the trial and consult the Advisory Group at key decision points.

### Composition of the data monitoring committee, its role, and reporting structure {21a}

There is no formal committee for the monitoring of data collected as part of this trial. Data monitoring will be undertaken by a researcher supervised by the trial biostatistician who will be removed from the daily activities of the trial. Questionnaire responses will be checked to determine any problems with the data collection instruments and any unforeseen negative impacts of participation.

### Adverse event reporting and harms {22}

The potential risks of participation in this trial are minimal. The workshops are already being delivered in schools by *Tomorrow Man*. *Tomorrow Man* facilitators are experienced in working with adolescents and are also trained in suicide prevention skills. They support participants during the workshop and then provide comprehensive handover of students to school teachers and/or welfare officers at the end of each group workshop. Participation in the trial will require students to complete two questionnaires focused on help-seeking, masculinity, and factors that are protective against suicide. It is not anticipated that completion of the questionnaire will produce any risks for participants greater than inconvenience or mild discomfort. In this trial, an adverse event will be defined as a participant becoming significantly distressed as a consequence of their involvement in the trial. If any adverse events are detected in the monitoring of the data, such as open-ended responses that indicate significant participant distress, then the Monash University Human Ethics Committee will be notified and they will advise the researchers about action to address the event.

### Frequency and plans for auditing trial conduct {23}

The principal investigator will meet at least fortnightly with researchers conducting the daily activities of the trial at the coordinating university to discuss and review trial progress, and at least monthly with the full investigator team. Any adverse events or issues in trial progress will be promptly communicated with the investigator team at these meetings or between meetings if necessary. The Advisory Group, comprising experts in male adolescent research and practice, will meet once per year for input into trial progress and will be contacted between meetings as needed. Progress to the trial funder will be reported annually. Any trial protocol amendments will be reported to the Australian and New Zealand Clinical Trials Registry (ANZCTR). No auditing will be undertaken.

### Plans for communicating important protocol amendments to relevant parties (e.g., trial participants, ethical committees) {25}

Any amendments to the protocol will be provided to the Australian New Zealand Clinical Trials Registry and will also be communicated to trial researchers and *Tomorrow Man*.

## Dissemination plans {31a}

Participating schools, students and parents, *Tomorrow Man*, and the funders will be provided with a summary of the findings of the trial. The findings will also be reported in journal articles and presented at scientific conferences and in public forums as requested.

## Discussion

With a focus on improving intentions to seek help by addressing harmful masculine norms, the *Breaking the Man Code* workshops show great promise as a school-based upstream suicide prevention intervention. However, little is known about the effectiveness of interventions such as these for adolescent boys. This trial will fill a gap in the knowledge that is critically needed to inform future interventions with boys and men.

There are some methodological challenges that may be encountered and that may be further complicated by the COVID-19 pandemic. Firstly, schools have been burdened by a need to rapidly adapt to a changing environment. In 2020 in Australia, schools shifted to online learning for students during periods of lockdown. As such, schools may now feel unwilling to take on new activities, such as involvement in research, as they conserve staff energy and resources to manage any further potential upheavals. Parents may likewise be feeling weary of requests for further school-related activities. Consistent with this supposition, recruiting participants for the Consumer Reference Panel in 2020 was unsuccessful. *Tomorrow Man* also adapted the workshop to online delivery during the trial design phase, which introduced a new stratification factor to the trial.

Ethics approvals have been delayed as committees prioritize applications related to COVID-19. This delay meant that when contact with schools begun in October 2020, while ethics approval had been obtained from Monash University’s Human Research Ethics Committee, Department of Education approvals, to undertake research in government schools was still underway. Therefore, only recruitment of independent schools, for whom further ethics approvals were not required, was possible. Approval from the Department of Education in New South Wales has since been received, and applications are underway for Western Australia and Victoria.

In order to mitigate against any potential challenges in working with schools due to COVID-19, the trial was expanded from an initial focus only on schools in Victoria to include schools in New South Wales and Western Australia. Expansion to other locations (South Australia, Queensland, and the Australia Capital Territory) will be considered if school numbers are lower than anticipated in 2021. Schools will be provided with up to A$800 to reimburse them for any administrative costs incurred due to participation in the trial. Further data collection in 2022 has been planned for, should the desired number of schools not be reached in 2021. The funders, Australian Rotary Health and the Medical Research Future Fund, are supportive of the challenges faced by researchers during the pandemic. The trial can be paused or extended if need be.

Despite these potential challenges, the trial is well placed to be completed as per the protocol. Fortunately, the trial design was undertaken as the COVID-19 pandemic unfolded, such that plans to mitigate the impact of the pandemic could be built into the protocol—the pool of potential schools has been increased from one state to three, reimbursement for schools is provided, and data collection into 2022 has been budgeted for. It is also likely that as time goes one school and parents will adapt to the challenges of schooling during a pandemic and may be more receptive to involvement in research.

A gendered approach to suicide prevention is well-overdue and school-based interventions have great potential to foster protective factors in young men. The findings of the trial will be used to improve the workshops for future participants and will also have international relevance to other people designing and implementing suicide prevention interventions for boys and men.

## Trial status

Protocol version 1, 1 April 2021

Recruitment beginning April 2021, to be completed October 2022.
